# ADC textural features in patients with single brain metastases improve clinical risk models

**DOI:** 10.1007/s10585-022-10160-z

**Published:** 2022-04-08

**Authors:** Martha Nowosielski, Georg Goebel, Sarah Iglseder, Ruth Steiger, Lukas Ritter, Daniel Stampfl, Johanna Heugenhauser, Johannes Kerschbaumer, Elke R. Gizewski, Christian F. Freyschlag, Guenther Stockhammer, Christoph Scherfler

**Affiliations:** 1 Department of Neurology, Medical University Innsbruck, Anichstrasse 35, A-6020 Innsbruck, Austria; 2 Department of Medical Statistics, Informatics and Health Economics, Medical University Innsbruck, Innsbruck, Austria; 3 Department of Neuroradiology, Medical University Innsbruck, Innsbruck, Austria; 4 Neuroimaging Research Core Facility, Medical University Innsbruck, Innsbruck, Austria; 5 Department of Neurosurgery, Medical University Innsbruck, Innsbruck, Austria

**Keywords:** ADC maps, Textural features, Single brain metastases, Prognostic models

## Abstract

**Aims:**

In this retrospective study we performed a quantitative textural analysis of apparant diffusion coefficient (ADC) images derived from diffusion weighted MRI (DW-MRI) of single brain metastases (BM) patients from different primary tumors and tested whether these imaging parameters may improve established clinical risk models.

**Methods:**

We identified 87 patients with single BM who had a DW-MRI at initial diagnosis. Applying image segmentation, volumes of contrast-enhanced lesions in T1 sequences, hyperintense T2 lesions (peritumoral border zone (T2PZ)) and tumor-free gray and white matter compartment (GMWMC) were generated and registered to corresponding ADC maps. ADC textural parameters were generated and a linear backward regression model was applied selecting imaging features in association with survival. A cox proportional hazard model with backward regression was fitted for the clinical prognostic models (diagnosis-specific graded prognostic assessment score (DS-GPA) and the recursive partitioning analysis (RPA)) including these imaging features.

**Results:**

Thirty ADC textural parameters were generated and linear backward regression identified eight independent imaging parameters which in combination predicted survival. Five ADC texture features derived from T2PZ, the volume of the T2PZ, the normalized mean ADC of the GMWMC as well as the mean ADC slope of T2PZ. A cox backward regression including the DS-GPA, RPA and these eight parameters identified two MRI features which improved the two risk scores (HR = 1.14 [1.05;1.24] for normalized mean ADC GMWMC and HR = 0.87 [0.77;0.97]) for ADC 3D kurtosis of the T2PZ.)

**Conclusions:**

Textural analysis of ADC maps in patients with single brain metastases improved established clinical risk models. These findings may aid to better understand the pathogenesis of BM and may allow selection of patients for new treatment options.

**Supplementary Information:**

The online version contains supplementary material available at 10.1007/s10585-022-10160-z.

## Originality and Presentations

The authors confirm the originality of this study.

## Introduction

In patients with systemic malignancies, brain metastases (BMs) are a common complication affecting around 20% of patients [1]. Despite multidisciplinary treatment including surgery, irradiation and/or systemic treatment [2] BMs are associated with high morbidity and mortality [3, 4]. Until the advent of cancer immunotherapies in 2015, imaging studies on patients with brain metastases (BM) have been scarse. However, recent encouraging results that demonstrated intracranial responses of immunotherapy in patients with BM [5], have refreshed the field of BM research. So far, cerebral magnetic resonance imaging (MRI) investigations contributed to the prognostic assessment by mainly identifying the number of BM [6]. To this end, MRI texture parameters have not been integrated in established clinical prognostic scores in patients with BMs [7, 8].

Diffusion weighted imaging (DWI) is a rapidly obtained and broadly available MRI sequence in clinical practice and is an integral part of standard brain tumor imaging [9]. It is able to yield ultra-structural information on cellular density [10] and properties of the extracellular matrix [11, 12] and has been linked to lesion aggressiveness and tumor response [13]. The mean apparent diffusion coefficient (ADC) in BM correlated with survival and recurrence after surgical resection [12, 14] and survival after radiosurgery [15]. Lately it could be also shown that mean ADC in the tumor core improved clinial risk models [14] and mean ADC changes at the tumor edge indicated a more locally aggressive phenotype [16, 17].

Texture analysis (TA) attempts to provide a non-invasive comprehensive quantitative analysis of image heterogeneity [18, 19]. A statistical based modelling is utilized involving three orders of measure parameters; first-order statistics summarize voxel values of a dedicated region of interest and report on descriptive parameters such as means and deviations, second-order statistics explore via co-occurrence measurements the length of voxels consecutively that have equal grey-level intensities, e.g. fine texture will have shorter lengths and a more consistent range of intensities and higher-order statistics explore the overall differences between pixels or voxels within the context of the entire region of interest. Neurooncologic studies indicated that TA has the potential to outperforme clinical and radiologic risk models in predicting prognosis e.g. in glioblastoma patients [20]. Recently TA of T1 and T2 weighted images has shown to allow a classification of BM by their primary site of origin [21].

In this study we retrospectively performed a textural analysis of ADC images derived from DWI in patients with single brain metastases in order to investigate its potential to improve established clinical risk models.

## Methods

### Study design, setting, participants

This was a retrospective study in a single center of 87 adult patients with single brain metastases from different primary tumors over a 15 year period from 2000 to 2015. The retrospective data analysis was approved by the local ethics committee of Innsbruck Medical University. Clinical as well as histological data were obtained by retrospective chart review and are detailed in Table 1. In this time period all cases with a solitary brain metastasis and sufficient MRI data (T1 weighted imaging, with and without contrast, T2 or FLAIR weighted imaging and DWI including ADC maps) were included. Patients who had radiation therapy, either locally or whole brain, and surgery prior to study enrolment were excluded as this could have altered the diffusion characteristics. Patients were excluded with single metastases of a diameter of ≤ 1 cm as the textural parameter as well as the ADC maps analysis could be affected by partial volume effects [22]. Only patients with a histological diagnosis of the primary tumor were included.

**Table 1:** Patient characteristics

	Cohort (n = 87)	
**Variable**	**Median**	**IQ range**
age	61.7	14.48
	**Category**	**Count (% of count)**
gender	female	56 (64.4)
	male	31 (35.6)
primary cancer	lung	46 (52.9)
	melanoma	5 (5.7)
	breast	5 (5.7)
	kidney	5 (5.7)
	GI cancer	9 (10.3)
	other	17 (19.5)
KPS	> 70	69 (79.3)
	< 70	18 (20.7)
systemic disease status	progressive disease	51 (58.6)
	stable disease	27 (31.0)
	partial/complete response	5 (5.7)
	unknown	4 (4.6)
presence of extracranial metastases	yes	62 (71.3)
	no	24 (27.6)
	unknown	1 (1.1)
RPA class	I	22 (25.3)
	II	46 (52.9)
	III	18 (20.7)
	unknown	1 (1.1)
DS-GPA score	0-1.5	10 (11.5)
	2.0-2.5	41 (47.1)
	3.0–4.0	22 (25.3)
	no category	14 (16.1)
surgery	yes	65 (74.1)
	no	22 (25.3)
stereotactic radiosurgery	yes	21 (24.1)
	no	64 (73.6)
	unknown	2 (2.3)
WBRT	yes	59 (67.8)
	no	21 (24.3)
	incomplete	5 (5.7)
	unknown	2 (2.3)
adjuvant chemotherapy	yes	6 (6.9)
	no	80 (92.0)
	unknown	1 (1.1)

KPS = Karnofsky performance status, RPA = recursive partitioning analysis, DS-GPA = disease specific graded prognostic assessment, IQ range = interquartile range, WBRT = whole brain radio therapy

### Imaging acquisition

Patients have been investigated on different scanners using 3T and 1,5T (Siemens Symphony Vision, Siemens Symphony Tim, Siemens Avanto, Siemens Sonata, Siemens Verio, Siemens Skyra). Importantly, diffusion weighting was applied with b-values at 0 and 1000 s/mm^2^ in all patients. For details on the imaging protocol, please see Supplement 1.

### MRI processing

Individual 3D T1 weighted MRI were segmented into gray matter, white matter and cerebrospinal fluid (CSF) compartments using statistical parametric mapping (SPM, Wellcome Department of Cognitive Neurology, London, United Kingdom). To compensate for eddy currents, DWI images were registered to an individual reference image without diffusion weighting. Registered DWI were visually verified for correct calculation and reconstruction for every subject. In order to standardize ADC values among MRI scanners, previously delineated areas of the tumor and edema as well as the compartment of the CSF were deduced from the gray and white matter compartments. Consecutively, the ratio of ADC values of each individual voxel within the tumor respectively within the edema and the mean ADC value of the tumor-free compartment was calculated. In order to avoid contamination from CSF and non brain compartments due to partial volume effects, ADC voxel values that were outside a threshold of mean CSF ADC of 2SD (standard deviations), determined for each individual, were excluded.

### Image segmentation and registration

**Fig. 1:**
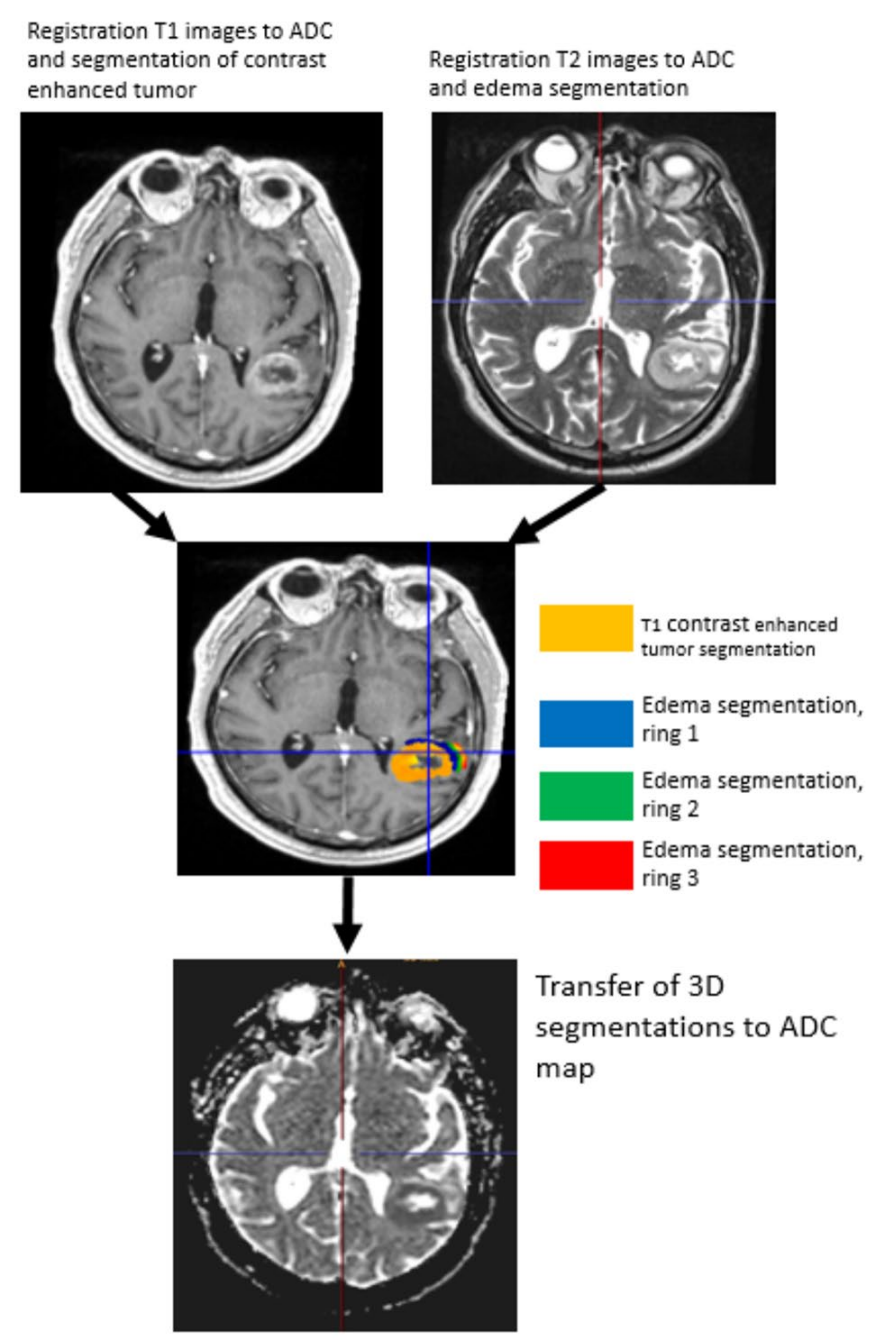
Segmentation, registration of T1 contrast enhancing MR images as well as T2 images zo corresponding apparent diffusion coefficient (ADC) maps. We subdivided the peritumoral space into three adjacent ring-shaped spaces with an orthogonal diameter of each 3 mm and calculated the mean ADC of each ring as well as the ADC slope from the outer to the inner ring

T1 weighted images as well as T2/FLAIR weighted images were co-registered to the corresponding ADC map sequence by using the software package statistical parametric mapping (SPM) [23]. Tumor segmentation was done by one person (LR) using a semi-automated active contour method (ITK-SNAP 2.0), which demonstrated excellent reliability and high efficiency of 3D segmentation [24]. The contrast-enhancing region in T1 weighted sequences, the non-enhancing T2 hyperintense region in T2/FLAIR weighted sequences, defined as peritumoral region (T2PZ) as well as the tumor- and edema-free gray and white matter compartment (GMWMC) were selected, representing three regions of interest. Contrast enhancing tumor regions and necrotic areas were excluded from the T2PZ. Within these three regions different ADC parameters were calculated using both MAtlab for first order and volumetric features and MaZda software package for textural features [25]. For a list of first order, volumetric and textural features please see Supplement 2. As studies have shown that the ADC may differ within the inner and outer border of the peritumoral region [17], we subdivided the peritumoral space into three adjacent ring-shaped spaces with an orthogonal diameter of each 3 mm, calculated the mean ADC of each ring Fig. 1.

### Textural Analysis

Altogether thirty imaging parameters were generated including first order features (normalized mean ADC and normalized 5% ADC in the contrast enhancing region, the T2PZ including the 3 rings separately as well as in the GMWMC), volumetric features (contrast enhancing region and T2PZ), as well as ADC textural features in the T2PZ. To calculate the change of the ADC within the outer and the inner ring, the parameter ADC slope was calculated.

### Outcome parameters

Overall survival (OS) was calculated from initial diagnosis of the brain metastasis until death, censored at the last recorded clinical contact and last follow-up (May 15th 2020). The most widely used clinical scores, the DS-GPA and the RPA, for predicting survival were calculated retrospectively using the clinical chart information. Supplement Table 3 for indivivual parameters.

### Statistical methods

Median with 95% Conficence Intervals [in brackets] are provided for all parameters. In univariate analysis differences in OS were calculated using Kaplan Meier analysis and log rank test, based on each of the factor listed in Table 1 and the individual imaging features. To test for the association of a combination of imaging parameters on survival, a linear backward regression model was used for selection of imaging features (dependent variable: log survival time for deceased patients). A cox proportional hazard model with backward regression was fitted for the established clinical prognostic models (DS-GPA and RPA) separately and then included the remaining imaging features to test for association of the imaging features on existing prognostic models. The goal was to examine which model best described overall survival. To compare the best fitting Cox proportional hazard models Akaike’s Information criterion (AIC) was used, which is a way of measuring concordance (how well the model fits the data) [26]. Low AIC value indicates a more accurate model. Statistical analysis was performed with SPSS 26.0 and Stata 13.0.

## Results

### Clinical outcome

Median age at diagnosis was 61.87 years [59.53–64.31]. Median OS was 9.48 months [17.41–31.52]. A total of 75 deaths were observed in our cohort (86.2%) during a median follow up of 10.51 years, minimum follow up 5.31 years, maximum follow up 20.22 years.

Older age at diagnosis (hazard ratio [HR] for death = 2.16, [1.35;3.44], p = 0.001, worse Karnofsky performance status (KPS, HR = 2.35, [1.3;4.1], p = 0.006), presence of extracranial metastases (HR = 1.67, [0.97;2.87], p = 0.05), systemic disease status (partial response and stable disease vs. progressive disease, HR = 3.05, [0.98 vs. 9.98], p = 0.001) were significantly associated with shorter survival in univariate analysis. A univariate cox model with cancer type as the independent predictor did not show a significant relationship between cancer type and time to death (p = 0.067).

Diagnosis specific GPA ((0-1.5) group I vs. (2.0-2.5) group II vs. (3.0–4.0) group III) revealed a HR for death = 5.90 (group I vs. III), [2.47;1.05] and a HR = 2.06 [1.14;3.75]) between group I and II (all p = 0.001).

RPA categories were also signficantly associated with differences in survival using a univariate cox model (I vs. II; HR = 2.58, [1.43;4.65], group I vs. III HR = 3.93, [1.92;8.04]).

Adjuvant WBRT was administered in 59 (67,8%) patients and was associated with significant longer survival (HR = 1.70, [1.04;2.84], p = 0.03). Neither surgery of the brain metastasis (p = 0.06) nor stereotactic radiosurgery (p = 0.42) nor adjuvant chemotherapie (p = 0.88) were associated with overall survival.

### Imaging biomarker and influence on survival and clinical models

Imaging features, individually, were not associated with survival, however linear backward regression identified eight independent imaging parameters which in combination were significantly associated with survival. These features were derived from different imaging sequences and regions of interest and included first order features and texture features from gray level co-occurrence and one volumetric feature, Table 2.

**Table 2:** Imaging Parameters significantly associated with survival

ADC texture features from T2PZ
- 3D skewness
- 3D kurtosis
- Mean contrast
- Mean entropy
- Mean difference in entropy
Volume of T2PZ
Normalized mean ADC of GMWMC
Mean ADC slope of the 3 rings in T2PZ

T2PZ = peritumoral border zone, GMWMC = gray matter white matter compartment, ADC = apparent diffusion coefficient

To test whether these imaging parameters may improve existing clinical prognostic models (DS-GPA and RPA) a cox backward regression for the established clinical scores DS-GPA and RPA including these 8 radiological features was calculated. Two MRI features remained -the normalized mean ADC of GMWMC and 3D kurtosis of ADC in T2PZ- and showed to improve the two prognostic models (HR = 1.14 [1.05;1.24], p = 0.003) for ADC GMWMC and HR = 0.87 [0.77;0.97], p = 0.018) for 3D kurtosis in T2PZ.

To compare the best fitting Cox proportional hazard models Akaike’s Information criterion (AIC) was used. The DS-GPA alone yielded an AIC of 476.78 which was decreased by the two imaging parameters to 470.13. Similarly, the RPA alone showed an AIC of 585.88 which was improved by the imaging parameters to 581.60.

## Discussion

In this retrospective study we investigated different brain and tumor compartments in patients with single BM by ADC texture analysis. We could show that eight independent imaging parameters (5 ADC textural features generated within the peritumoral region, the volume of the peritumoral region, the normalized mean ADC in the GMWMC as well as the mean ADC slope within the peritumoral region) predict survival. Furthermore, in Cox regression analysis, the mean ADC of the GMWMC as well as 3D kurtosis, a textural ADC feature of the peritumoral border zone, improved the two established clinical risk models, the RPA and the DS-GPA.

Assessment of prognostic and predictive biomarkers is a major goal in neurooncologic studies for better risk stratification and prediction of response to treatment. Many clinical scales exist for predicting survival in patients with BM [7, 27]. The RPA score and the DS-GPA score are the most frequently used scores to guide treatment decisions in BM [8, 27, 28]. Clinical parameters that are integrated into these scores are the KPS, age of the patient, type of the primary tumor, number of BM and the presence or absence of extracranial metastases. In our retrospective study design over a 15 year period we could confirm that the RPA as well as the DS-GPA predict survival. Older age at diagnosis, worse KPS, presence of extracranial metastases and systemic disease status were significantly associated with shorter survival in our analysis. Interestingly neither cancer type, surgery, stereotactic radiotherapy nor adjuvant chemotherapy were shown to be an independent predictor for survival allowing us to include all tumor types into further radiological analysis. Recently also molecular parameters were included into the scores to account for the markedly heterogenous population of patients with BM [7, 8, 29–36]. Due to the retrospective setting of our study, however, we could not include any of these molecular markers.

Currently, except for the number of BM no non-invasive biomarker is included into the prognostic scores. Imaging may overcome the heterogeneity in space which often limits molecular diagnostics. By quantitatively analysing the different brain and tumor compartments in patients with single BM we could show that two imaging parameters derived from ADC maps contribute to a better prognostication when put into a cox regression model together with the established clinical scores. The first paramater was the normalized mean ADC of the tumor-and edema-free surrounding brain tissue (GMWMC). This region represents the presumambly “healthy” and “non-affected” surrounding gray and white matter compartment of the patients excluding contrast enhancing tumor, peritumoral T2 hyperintensities, necrotic areas, ventricles and sulci including CSF. The changes of the normalized mean ADC in this region are very subtle. The GMWMC parameter showed a HR of 1.14 to improve the prognostic model. This means that a 1/100 increase of the mean ADC was associated with a 14% higher risk of dying. Higher ADC values have shown to be associated with a decreased extracellular matrix density [12] and a greater degree of tumor differentiation [16] in BM. Changes of the white and gray matter compartment and associations with survival have not been reported so far in patients with BM. These data warrant further research to identify altered ADC values in the healthy surrounding brain to predict development of new BM. This might help to guide treatment (e.g. radiotherapy, whole brain radiotherapy versus stereotactic treatment) in a more personalized way.

A multidisciplinary study recently investigated the mean ADC within the contrast enhancing tumor part in patients with single BMs showing that the mean ADC improves the prediction of the RPA as well as the GPA [14]. Prior studies investigating ADC in patients with BM have shown that patients with small peritumoral edema have shorter survival times and their tumors were characterized by a more brain-invasive growth, lower HIF1a expression and less angiogenic activity [17]. Similarly, the changes in diffusion across the tumor border and in peritumoral brain tissue were associated with survival. It could be shown that BM with a sharp change in diffusion across their border showed shorter overall survival compared to those with a more diffuse edge [16]. Our study supports these findings by showing that the volume of the peritumoral edema as well as the ADC slope, which is a good quantitative parameter to detect changes of ADC at the peritumoral border, were among the 8 radiologic parameters that were associated with survival in linear backward regression analysis.

In addition to the prognostic role of the GMWMC we identfied 3D kurtosis, a textural parameter of the peritumoral edema as a second prognostic factor which improved the two clinical scores. 3D kurtosis is a measure of the tailedness of values and describes the shape of a probability distribution. The higher the kurtosis, hence the more peaked the distribution of the ADC values in the peritumoral edema was, the lower was the survival in our study (HR 0.87). A peaked curve also implies a more homogeneous ADC value distribution. Studies on tumor-infiltrating lymphocytes (TILs) in BM showed that the density of TILs correlated positively with the extent of peritumoral edema and showed a positive correlation with favorable median OS [37]. It might be hypothesized that TILs cause a change in the textural composition of the peritumoral region. The more TILs, the more heterogenous the ADC distribution might get. In context with these findings, we hypothesize that the 3D kurtosis of the peritumoral edema may reflect the amount of TILs and might serve as a potential biomarker for immunotherapy in cancer patients affecting the CNS.

In a retrospective study [38] of 88 patients treated by immunotherapy due to melanoma BM, T1 contrast enhanced lesions were investigated by radiomic analysis in order to detect predictive biomarkers for survival. Multiple features were associated with increased overall survival, however in multivariate analysis no significant association with survival could be detected. In this context ADC analysis may be more useful, because in contrast to T1 and T2 weighted imaging, ADC values are quantitative parameters allowing for good comparisons between scanners. Entropy values of ADC maps derived from DWI consistently showed promising results for differentiating low-grade gliomas from high-grade gliomas [39, 40]. 3D TA appears also more accurate than 2D, given the high spatial resolution of the acquired data. Similarly, results based on a volumetric analysis appear more reliable than those based on a single slice [41]. Despite the heterogeneity of the data and software available, most studies demonstrate the robustness of the texture analysis and its clinical transferability for diagnostic use [41]. TA on DTI-derived fractional anisotropy and ADC maps showed significantly higher heterogeneity in peritumoral edema of glioblastomas compared with metastases differentiating them with a sensitivity of 80% and specificity of 90% [42].

A major limitation of this study is the lacking of a validation cohort. However, the statistical model used in our study is conservatively chosen in order not to overfit the data existing data. Backward regression is a stepwise regression approach that begins with a full model (including all data) and at each step gradually eliminates variables from the regression model to find a reduced model that best explains the data (inour case the DS-GPA and RPA) [43, 44]. The stepwise approach is useful because it reduces the number of predictors, reducing the multicollinearity problem and it is one of the ways to resolve the overfitting. By this approach we reduced the number of imaging parameters to identify the imaging features that really added value to the prognostic models, DS-GPA and RPA. A second limitation is the lacking of molecular parameters. Until recently, Sperdutos [7, 8] group has published a series of articles regarding diagnosis-specific prognostic factors including also molecular parameters for the markedly heterogenous population of patients with BM, e.g. HER2 and estrogen/progesterone receptor status in breast cancer [29], BRAF mutation in melanoma [32, 33] or EGFR and Alk status in NSCLC [30, 31]. Unfortunately, these parameters were not available in our retrospective patient cohort.

In conclusion, we could show that ADC textural features of different brain and tumor compartments in patients with single BM improve established clinical risk models. The radiologic characterization of the peritumoral region as well as the region of the surrounding brain tissue might help to guide treatment at first diagnosis of the disease, allowing for better prognostication and earlier detection of new BM. In addition, these findings might add to better assess response of targeted therapies and immunomodulatory therapies.

## Electronic Supplementary Material

Below is the link to the electronic supplementary material.

Supplementary Material 1

## Data Availability

data will be made available on reasonable request.

## Abbreviations

ADC: apparent diffusion coefficient.
BM: brain metastases.
DW-MRI: Diffusion weighted MRI.
T2PZ: T2 peritumoral borderzone.
GMWMC: gray matter white matter compartment.
MRI: Magnetic Resonance Imaging.
DS-GPA: diagnosis-specific graded prognostic assessment score.
RPA: recursive partitioning analysis.
TA: texture analysis.
2SD: 2 standard deviations.
CSF: cerebrospinal fluid.
