# Supplementary material for: ADC textural features in patients with single brain metastases improve clinical risk models
Source: Clin Exp Metastasis. 2022 Apr 8;39(3):459–66. doi: 10.1007/s10585-022-10160-z (PMC9117356; doi:10.1007/s10585-022-10160-z)

**Data supplement for**

**Textural features of ADC maps in patients with single brain metastases improve clinical risk models**

Corresponding author:

Martha.nowosielski@i-med.ac.at

**Content:**

**Supplement Table1 MRI sequence parameters**

**Supplement Table 2 MRI radiomic parameters**

**Supplement Table 3 Outcome parameters**

# *MRI sequence parameters*

**Table 1.**

| Scanner Type | Number of subjects | Tesla | T1-weighted MRI | | | | DTI | | | | | |
| --- | --- | --- | --- | --- | --- | --- | --- | --- | --- | --- | --- | --- |
|  |  |  | TR | TE | Fa | Voxel size (mm) | TR | TE | Fa | Gradients | b-Values | Voxel size (mm) |
| Siemens Sonata | 32 | 1.5 | 564 | 14 | 90 | 0.7×0.7×5 | 3700 | 107 | 90 | 3 | 0, 1000 | 1.8×1.8×5 |
| Siemens Symphony Vision | 12 | 1.5 | 1710 | 4.38 | 15 | 0.9×0.9×5 | 6100 | 108 | 90 | 3 | 0, 1000 | 0.9×0.9×5 |
| Siemens Symphony Tim | 25 | 1.5 | 1830 | 3,62 | 15 | 0.9×0.9×1.2 | 3000 | 80 | 90 | 12 | 0, 1000 | 1.8×1.8×5 |
| Siemens Avanto | 10 | 1.5 | 1600 | 3.44 | 15 | 0.9×0.9×1.2 | 6000 | 94 | 90 | 12 | 0, 1000 | 0.9×0.9×3 |
| Siemens Verio | 3 | 3 | 1750 | 3.29 | 9 | 0.7×0.7×5 | 7500 | 95 | 90 | 20 | 0, 1000 | 0.9×0.9×3 |
| Siemens Skyra | 5 | 3 | 1820 | 2.96 | 8 | 0.8×0.8×1 | 7000 | 95 | 90 | 20 | 0, 1000 | 0.9×0.9×3 |

# *S2 MRI parameters*

We calculated 30 MRI parameters for each patient; 8 first-order features, 5 volumetric features and 17 ADC texture features.

**First order features**

- Normalized 5% percentile ADC peritumoral edema
- Normalized 5% percentile ADC contrast enhancing tumor
- Normalized mean ADC in peritumoral edema
- Normalized mean ADC in contrast enhancing tumor
- Normalized mean ADC in brain mask
- Normalized 5% percentile ADC in brain mask
- Normalized mean ADC in ring 1, ring 2, ring 3
- ADC slope between ring 1-3

**Volumetric features**

- Volume peritumoral oedema
- Volume contrast enhancing tumour
- Ratio oedema to contrast enhancing tumour (volume oedema/volume contrast enhancing tumour)
- Ratio contrast enhancing tumour to oedema (volume contrast enhancing tumour/volume oedema)
- Total volume (volume contrast enhancing tumour + volume oedema)

**Textural features (description see below)**

- Skewness3D
- Kurtosis3D
- Gradient Mean
- Gradient Variance
- Gradient Skewness
- Gradient Kurtosis
- Mean angular second moment energy (x1000)
- Mean contrast
- Mean correlation
- Mean sum of squares
- Mean Inverse Difference Moments
- Mean Sum Average
- Mean Sum Variance
- Mean Sum Entropy
- Mean Entropy
- Mean Difference Variance
- Mean Diffference Entropy

Texture parameters computed by MaZda (Szczypiński PM et al (2009) MaZda–a software package for

image texture analysis. Comput Methods Programs Biomed 94:66–76)

| Histogram | Absolute gradient | Co-occurrence matrix |
| --- | --- | --- |
| Mean ADC | mean | angular second moment |
| variance | variance | contrast |
|  |  | correlation |
|  |  | sum of squares |
| skewness | skewness | inverse difference moment |
| kurtosis | kurtosis | sum average |
|  |  | sum variance |
|  |  | sum entropy |
|  |  | entropy |
| 5^th^ percentile | percentage of pixels with nonzero gradient. | difference variance |
|  |  | difference entropy |

The distances d = 1, 2, 3, 4 and 5 pixels with angles q; = 0°, 45°, 90° and 135°were considered when generating the co-occurrence matrix. The values represent the mean of the directions and distances.

Definition and discussion of the above parameters can be found in (Haralick 1973, Haralick 1979, Hu 1994, Lerski 1993). A quick texture parameter reference is provided below for the user convenience.

To keep consistency with the formulas used in the standard references (Haralick 1973, Haralick 1979) on texture analysis, it is assumed in MaZda that the intensity of image under analysis changes from 1 to Ng, where Ng = 2k, and k is the number of bits per pixel. Thus if originally the image intensity changes from 0 to Ng-1, MaZda converts this image internally, such that its intensity changes from 1 to Ng. Consequently, the summation indices in the formulas listed below span the range from 1 to Ng.

**Histogram-based features**

In the formulas that follow, p(i) is a normalized histogram vector (i.e. histogram whose entries are divided by the total number of pixels in ROI), i=1,2,..., Ng, and Ng denotes the number of intensity levels.

Mean:
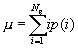


Variance:
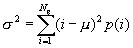


Skewness:
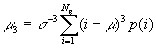


Kurtosis:
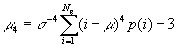


**Gradient-based parameters**

For the gradient feature calculation the following neighborhood for image pixel x(i,j) is defined:


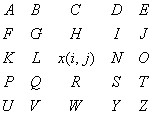


Based on this neighborhood, the absolute gradient value (ABSV(i,j)) is calculated for each pixel:

1. for 5x5 pixel neighborhood:
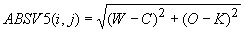

2. for 3x3 pixel neighborhood:
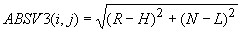


The ABSV3 definition is used in this version of MaZda. For the ABSV=ABSV3 matrix of M elements (which contains absolute gradient values for ROI pixels), the gradient features are defined as follows:

Mean absolute gradient:
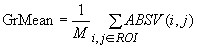


Variance of absolute gradient:
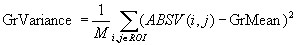


Skewness of absolute gradient:
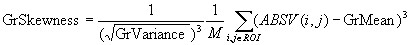


Kurtosis of absolute gradient:
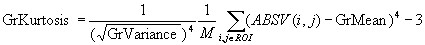


where ROI is a region of interest.

Percentage of non-zero ABSV matrix elements (Grads>0)

**Co-occurrence matrix-derived parameters**

The second-order histogram is defined as the co-occurrence matrix hdq(i,j) [1]. When divided by the total number of neighboring pixels R(d,q) in ROI, this matrix becomes the estimate of the joint probability, pdq (i,j), of two pixels, a distance d apart along a given direction q having particular (co-occurring) values i and j. Formally, given the image f(x,y) with a set of Ng discrete intensity levels, the matrix hdq(i,j) is defined such that its (i,j)th entry is equal to the number of times that and,


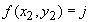

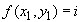


where
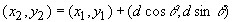
.

This yields a square matrix of dimension equal to the number of intensity levels in the image, for each distance d and orientation q;. In MaZda, the distances d = 1, 2, 3, 4 and 5 pixels with angles q; = 0°, 45°, 90° and 135° are considered. Reduction of the number of intensity levels (by quantization to fewer levels of intensity) helps increase the speed of computation, with some loss of textural information.

The co-occurrence matrix-derived parameters computed by MaZda are defined by the equations that follow, where mx, my and sx, sy denote the mean and standard deviations of the row and column sums of

the co-occurrence matrix, respectively [related to the marginal distributions px(i) and py(j)].

Angular second moment:
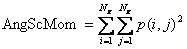


Contrast:
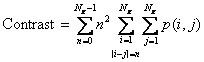


Correlation:
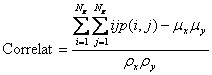


Sum of squares:
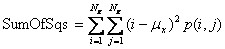


Inverse difference moment:
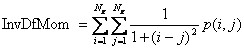


Sum average:
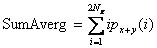


where


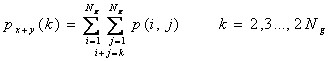


Sum variance:


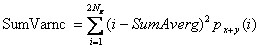


Sum entropy:


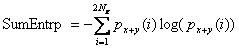


Entropy:


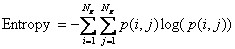


Difference variance:


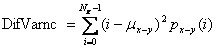


where m;x-y is a mean value of difference distribution px-y:


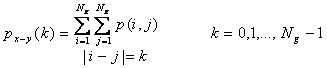


Difference entropy:


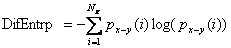


# *S3. Outcome parameters*

**DS-GPA (diagnosis-specific graded prognostic assessment score)**


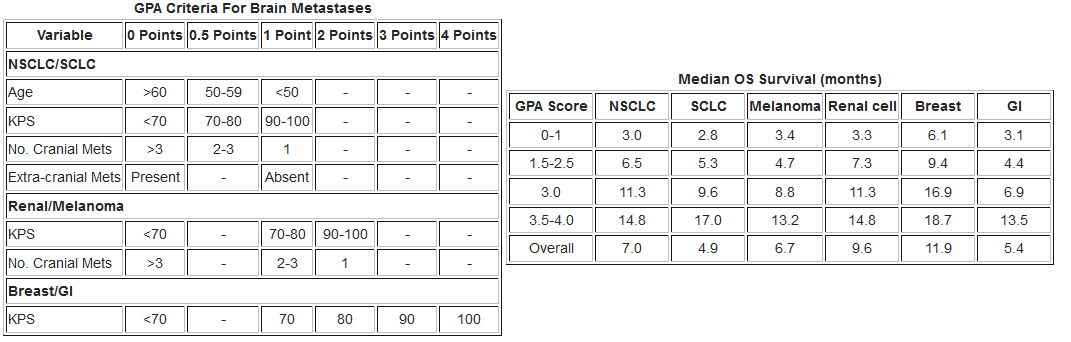


**RPA (Recursive partitioning analysis)**


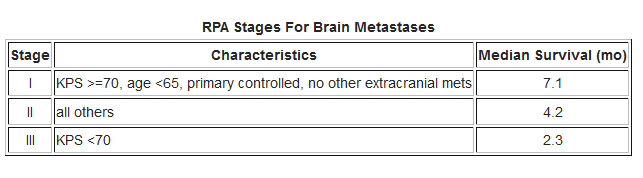

Supplement: Supplementary file 1 — Supplementary Material 1 [file 10585_2022_10160_MOESM1_ESM.docx]
